# A non-linear game for two: genetic parameters and prediction of fertilization success using Bayesian and machine learning frameworks

**DOI:** 10.1186/s12711-026-01070-9

**Published:** 2026-07-16

**Authors:** Fotis Pappas, Paul Vincent Debes, Martin Johnsson, Christos Palaiokostas

**Affiliations:** 1 Department of Animal Biosciences, Swedish University of Agricultural Sciences, Uppsala, Sweden; 2 Department of Aquaculture and Fish Biology, Hólar University, Sauðárkrókur, Iceland

## Abstract

Fertility is an important but often cryptic and intrinsic characteristic of domesticated animals. Predicting reproductive potential is of great importance for the industry but assessment through indirect proxies is laborious and often impractical. Among other biological factors, genetic effects are expected to play a crucial role in shaping male and female fertility. In cases where heritable components are strong, polygenic merit could be a valuable tool for decision-making in breeding schemes. Here we estimate sex-specific variance components affecting fertilization success by analyzing outcomes of over 3000 controlled mating events in an Arctic charr breeding nucleus from Iceland. Furthermore, a machine learning framework using relationships-to-founders vectors as input and a two-tower neural network architecture is proposed and tested for prediction of fertilization success. Both approaches seem to capture a meaningful biological signal and offer alternative tools for ranking, selecting or even allocating matings between breeding candidates.

**Supplementary Information:**

The online version contains supplementary material available at 10.1186/s12711-026-01070-9.

## Background

Reproductive success is a fundamental prerequisite of sustainable and profitable animal production. In salmonid farming, the relative economic value of each breeder is particularly high due to the high reproductive potential of these species [1, 2]. However, external fertilization is an environmentally sensitive process [3] and thus requires substantial investment in resources and labor. Combined with the typically narrow spawning windows especially for females [4], fertilization failure becomes extremely costly, leading not only to wasted resources but also to potential reductions in population size and genetic diversity. Ensuring consistent and reliable fertility is therefore critical to the long-term viability of breeding programs.

Traditionally, reproductive capacity in breeders has been assessed using proxy traits such as gamete counts and quality measures (e.g., egg size, sperm motility) [5]. While informative, the collection of such phenotypes is often impractical, given the associated costs and labor intensity. An interesting alternative is to predict fertility through biological predictors. More specifically, estimating the genetic merit for fertility in candidate breeders lacking own phenotype could simultaneously improve fertilization rates in the short term and drive long-term genetic gains by shifting the population mean through more effective selection enabled by minimizing fertility-related loss of families.

Interestingly, familial fertilization rates are governed by intrinsic biological factors contributed by (at least) two individuals acting in a non-additive manner. A probabilistic, multiplicative framework that accounts for the non-linear effects of sires and dams on familial fertilization rates may therefore provide the most realistic description of mating outcomes [6–8]. Among potential analytical approaches, machine learning offers a suitable means to efficiently model such complex interactions [9].

In this study, we analyze more than 3000 mating events from the Icelandic Arctic charr breeding program (Hólar University, Iceland) [10] focusing on the genetic variance components of latent male and female fertility, as well as the applicability of machine learning architectures for predicting fertilization success and failure based on genetic merit.

Our work introduces two key analytical novelties. First, we propose the use of relationships-to-founders as input features, offering an alternative to previously used data sources such as genotypic information, the full additive genetic relationship matrix (***A***) or its low-dimensional representations through eigenvector analysis [9, 11–15]. Second, we developed a two-tower multilayer perceptron (MLP) [16] architecture with probability multiplication fusion, specifically designed to predict fertilization outcomes.

## Methods

### Background information of the studied population—collected data

Fertilization success data were recorded over 17 years (2008–2024) from the Icelandic Arctic charr breeding nucleus in Hólar, northern Iceland [10]. Records included a total of 3248 artificial mating events between 1969 sires and 3016 dams. Males often contributed to two mating events whereas dams usually contributed to one. With the introduction of optimal contribution selection in the 2022 cohort, some sires and dams contributed to several families, while many contributed to only one. The number of viable fertilized eggs was recorded under substantial right-censoring with up to 400 eyed eggs counted per mating. For our analysis, a binary fertilization outcome was considered with at least 400 eyed eggs (the upper bound) being considered a success given that females typically spawn thousands of eggs. This conservative definition of failures reflects the operational maximum number of eggs kept for establishment of full-sib families in the breeding nucleus and corresponds to an overall success rate of approximately 88% across all years.

### Estimation of genetic parameters for latent fertility traits

**Fig. 1:**
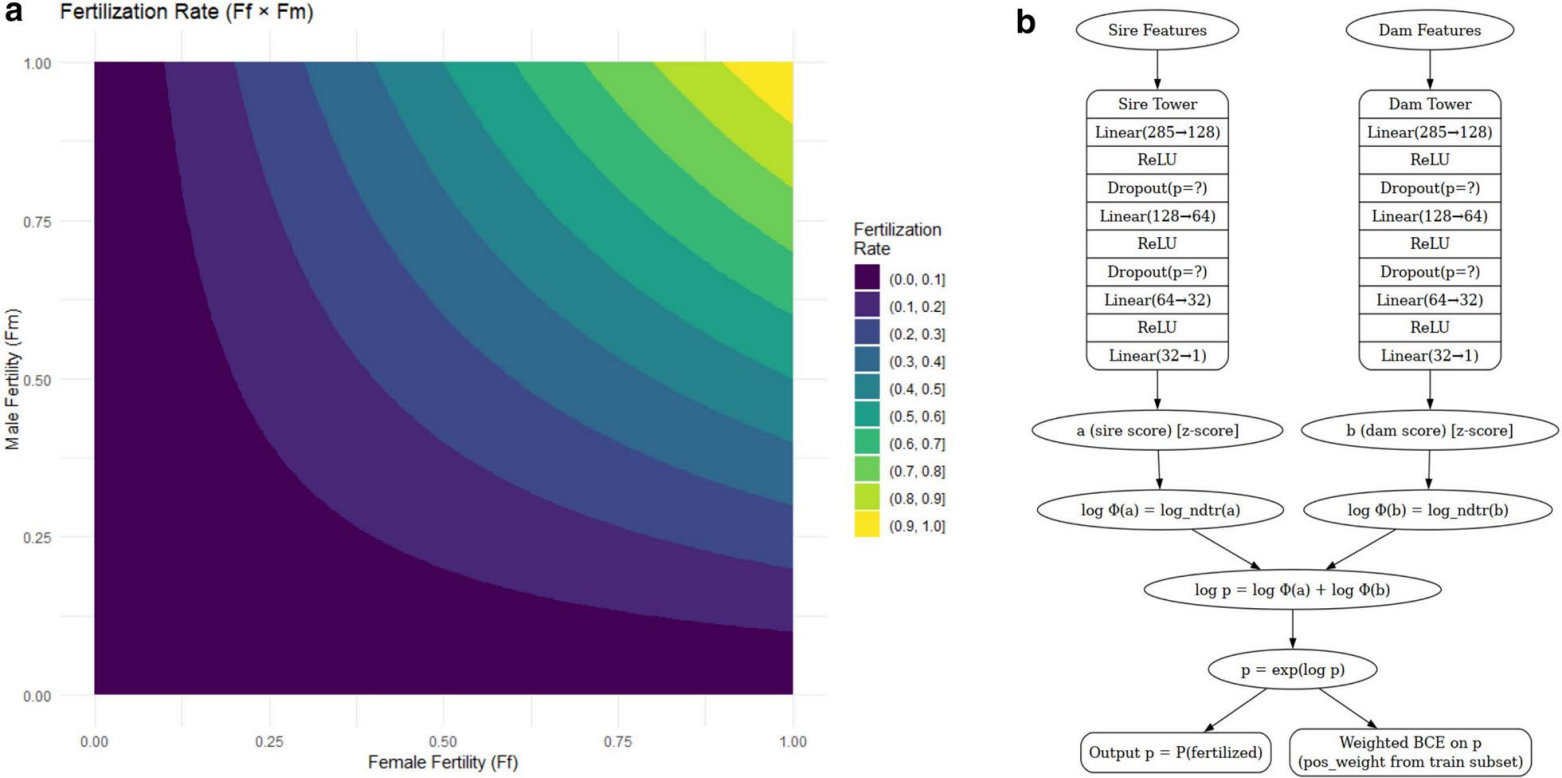
Conceptual foundations of computational frameworks.** a** Fertilization rate contours as a product of male (F*m*) and female (F*f*) fertility probabilities.** b** Two-tower MLP architecture with unknown drop rates for the first two hidden layers to be found through grid search. Each tower has different weights and outputs a liability score that is then transformed via a probit link to range [0, 1]. Fertilization probability is produced after multiplying the respective probabilities by exponentiating the sum of their natural logarithms for numerical stability. Created with ggplot2 v4.0.0 [27], graphviz v12.2.1 [28]

A sex-specific, bivariate model was constructed for the binary fertilization success trait discussed above using a Bayesian hierarchical framework after dropping records with phantom parents (3087 mating events retained). Under a Generalized Linear Mixed Model (GLMM) using probit-links, fertilization success requires both a female and a male latent liability to exceed zero. Each sex-limited, latent phenotype had its own intercept and year as random effects, while the additive genetic effects were modelled on the liability scale using the additive genetic relationship matrix ***A*** built with R/AGHmatrix [17, 18], utilizing the full pedigree with unphenotyped ancestors (total of 9219 entries). Additionally, a permanent-environmental effect was considered for sires, while both sexes shared a residual term that considered covariance. For mating event $i$, the observed fertilization outcome ${y}_{i}$ was coded 1 for success and 0 for failure, and modelled as:

$${y}_{i}=\mathrm{Bernoulli}({p}_{i})$$

where the success probability is defined as the product of the standard normal cumulative distribution function (CDF, denoted as $\Phi$) for female and male liabilities:

$${p}_{i}= \mathrm{Ff}_{i}\cdot \mathrm{Fm}_{i} =\Phi \left({\eta}_{\mathrm{female}_{i}}\right)\cdot\Phi \left({\eta}_{\mathrm{male}_{i}}\right)$$

where ${\eta}_{\mathrm{female}_{i}}$ and ${\eta}_{\mathrm{male}_{i}}$ are the latent fertility liabilities for the dam and sire of mating $i$ respectively (Fig. 1a visualizes a Fertilization Rate equivalent).

The model was implemented in Stan using the R/rstan package [17, 19]. We utilized the No-U-Turn Sampler (NUTS) [20], a variant of Hamiltonian Monte Carlo (HMC), with weakly informative priors. Overall, we run three chains 10,000 iterations each, with a 4000-iteration warmup phase. Convergence was assessed using $\widehat{R},$ effective sample size, and visual inspection of trace plots. Liability-scale additive variances, heritabilities [21], repeatability for male fertility and genetic correlation between male and female fertility were derived along with each animal’s estimated breeding values (EBVs).

### Two-tower MLP model

The same underlying multiplicative system, yielding fertilization success probabilities from female and male contributions was considered for our ML model. The proposed two-tower structure is inspired by widely adopted models in retrieval and recommendation systems first popularized by Google® for YouTube® recommendations [22].

For input, we define ***A***_***F***_ as the submatrix corresponding to relationships between all individuals and the pedigree founders (N = 285, including meta-founders). For each individual $i$, the row vector ${\mathbf{a}}_{i}$ represents its additive genetic relationships with all founders and was used as input (Fig. 1b) to sire- and dam-specific multilayer perceptron towers. Conceptually, this representation reflects expected identity-by-descent (IBD) allele sharing with the base population rather than direct pairwise relationships among relatives. This effectively reduces dimensionality compared to using the full relationship matrix ***A***, improving the ratio of input features to number of observations. Also, this type of input might help control overfitting, since predictions are driven by shared founder contributions instead of memorization of highly related individuals.

The architecture of each MLP—tower consisted of three hidden layers each, with rectified linear unit (ReLU) activations (architecture described in Fig. 1b). Training was carried out using the Adam optimizer [23] and early stopping to control training time and overfitting. Early stopping was applied by monitoring validation ROC-AUC on validation subsets generated within the training partition. Training was terminated after 15 epochs without improvement and restoring the model weights from the epoch with the highest validation ROC-AUC. Finally, a class-weighted binary cross-entropy (BCE) loss function was used for model training in order to account for class imbalance. During training, class weights were computed for the training data, so that errors on the minority class had a proportionally larger effect on the loss function. Model evaluation was performed on the unweighted test data. The neural networks were implemented in PyTorch [24], while cross-validation and evaluation metrics (ROC AUC, accuracy, Precision-Recall AUC and F1-score) were computed using the scikit-learn library in Python [25].

Observations were partitioned using stratification based on spawning year to prevent parent leakage (i.e., we avoid having the same breeder present in both training and validation/testing sets), combined with target stratification to maintain class balance (Table 1). An initial 80–20% split was performed to separate a dataset for fourfold cross-validation (80%, used for dropout tuning) and a final test-set partition (20%). Two dropout steps for the first two hidden layers were included with rates ranging between 0.0 and 0.5 evaluated via grid-search in the cross-validation phase of our analysis, meaning that between 0 and 50% of the neurons in these hidden layers were randomly disabled during training as a means of regularization. Final model training was then performed using the selected hyperparameters, and predictive performance was assessed on the independent 20% test partition.

**Table 1:** Summary of training and test set properties with number of crossed, dams, sires and success rate per spawning year

Spawning year	Number of crosses	Number of dams	Number of sires	Success rate (%)
*Training set*				
2008	188	188	99	95.74
2010	220	220	148	92.27
2011	226	226	119	98.23
2013	190	190	102	92.63
2014	155	154	118	91.61
2016	210	207	146	76.67
2017	157	157	136	80.89
2019	160	160	91	80.00
2020	203	203	126	79.80
2021	196	196	119	80.10
2022	205	168	102	93.66
2023	178	77	82	78.65
2024	161	82	108	93.17
Total	2449	2218	1485	87.38
*Final test set*				
2009	220	220	133	94.55
2012	152	152	94	95.39
2015	210	210	139	90.48
2018	217	217	118	84.79
Total	799	799	484	90.99

## Results and discussion

### Genetic parameters of latent fertility factors

Heritability estimates were 0.36 ([0.20, 0.56] 95% CI) for female fertility and 0.15 ([0.00, 0.43] 95% CI) for male fertility (trace-plots in Additional file 1 Fig. S1 and diagnostics in Additional file 2 Table S1) on the respective latent scales. The posterior mean for genetic correlation between the two traits was 0.14 but was uncertain ([– 0.64, 0.78] 95% CI). Repeatability posterior mean for male reproductive potential was 0.38 ([0.14, 0.62] 95% CI). Besides more heritable, female liability had a slightly higher point estimate of phenotypic variance (posterior mean = 2.80, [1.74, 4.96] 95% CI) than male liability (posterior mean = 2.25, [1.20, 4.56] 95% CI), although credible intervals overlapped substantially. Intercept estimates were somewhat higher for males (Additional file 2 Table S1). Compiled, these patterns provide limited evidence that dam fertility may be more variable, but considerable uncertainty remains. Furthermore, the heritability posteriors ranged at similar levels, but with an opposing sex-specific pattern compared to a recent study in the Swedish National Breeding Program, where male fertility heritability was higher than that of females, although the trait definitions differed considerably [8]. At the same time the posteriors in the current study appear narrower, yielding more confident parameter estimates, probably reflecting the larger sample size.

### Tuning and performance of two-tower MLP

**Fig. 2:**
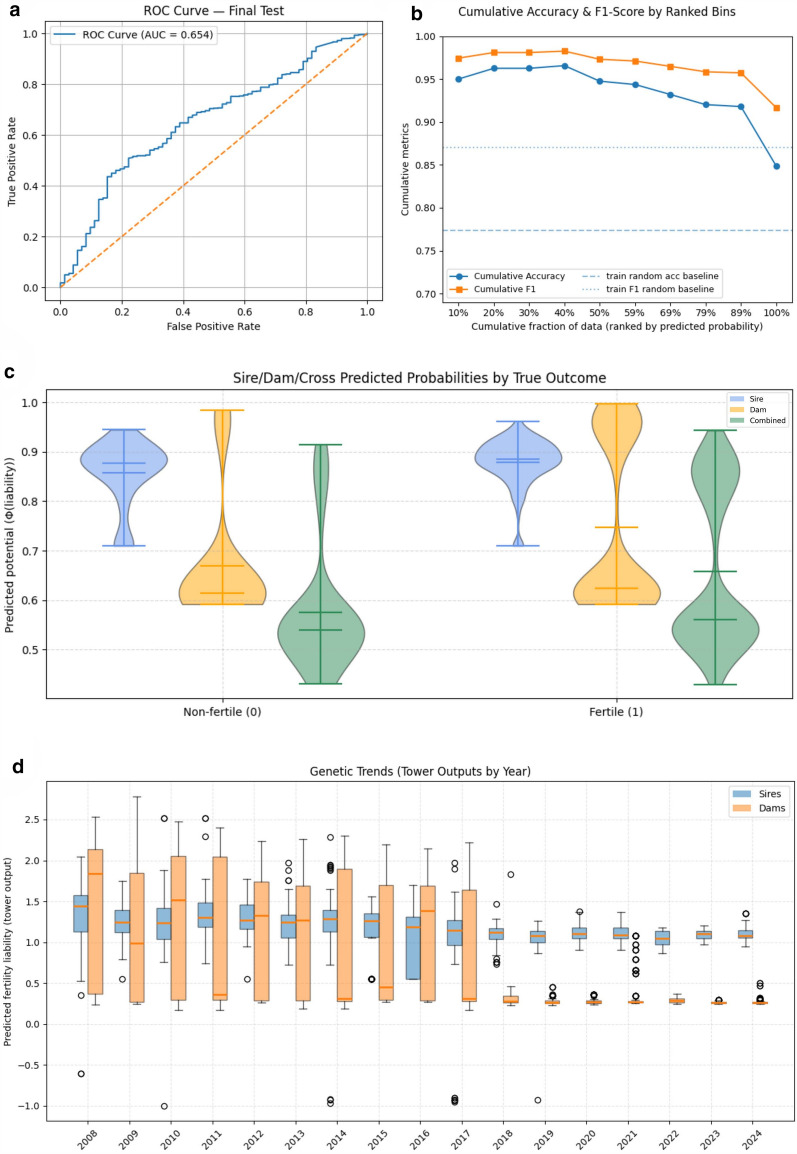
Two-tower model performance and predictions.** a** ROC plot (held-out test set) of the classifier based on the two-tower architecture,** b** cumulative accuracy and F1-score (held-out test set) by ranked fractions of data with baseline metrics corresponding to the training data,** c** violin plot of predicted reproductive potentials of sires, dams and corresponding pairs by true label in the held-out test set,** d** boxplot of sex-specific predictions (on whole dataset) by cohort acting as a proxy assessment of genetic trends. Created with matplotlib v3.10.5 [29]

Grid-search indicated dropout rates of 0.0 and 0.5 as the best-performing values for the first and second hidden layers, respectively. This configuration yielded an average ROC AUC of 0.645 across the four cross-validation folds (Additional file 3 Table S2), indicating that stronger regularization was beneficial in the second hidden layer. Overall, training with this architecture achieved an ROC AUC of 0.654 (Fig. 2a) and a PR-AUC of 0.945 on the final held-out test set. These results represent a 30.8% and 3.85% respective relative lift over the random (0.50) and prevalence-based (0.91 in test-set) baselines, reflecting notable discriminative power in a context where high success rate leaves little margin for improvement in precision. From a practical breeding management standpoint, when selecting the top 40% predicted outcomes, the expected accuracy was the highest (Fig. 2b). Given the low (sire) to moderate (dam) heritability of the traits, these predictive values are encouraging, even if the trait definition was not optimal. Importantly, the relative gain compared to random classification is valuable in a breeding context, where false positives can result in inefficient on-farm selection and misallocation of resources.

Unfortunately, the exact signals and interactions offering predictive ability to the model remain cryptic since the real fertility status of individual breeders is naturally masked. However, the tower outputs seem to generally be linked to true combined labels (Fig. 2c). Variation in liability outputs drops notably for more recent generations (Fig. 2d). This probably happens because expected additive relationships with founders become increasingly diluted across generations, leading to dense vectors of small founder contributions rather than a fewer but strong ancestral signals.

An additional strength of the two-tower design is its flexibility. Different data types and dimensionalities can be assigned to the male and female towers. For instance, female inputs could consist of SNP genotypes while male inputs might comprise sperm CpG methylation markers [26], enabling the model to integrate heterogeneous molecular signals. This makes the architecture adaptable and multi-purpose across species and data modalities. Furthermore, mate compatibility could also be modelled through more complex architectures resulting in deeper refinement of the biological background. Finally, if combined with interpretability techniques, the framework could also be employed for feature selection and biomarker discovery, helping to identify molecular signatures of fertility and infertility.

### Relationship of proofs from hierarchical Bayesian framework and two-tower MLPs

**Fig. 3:**
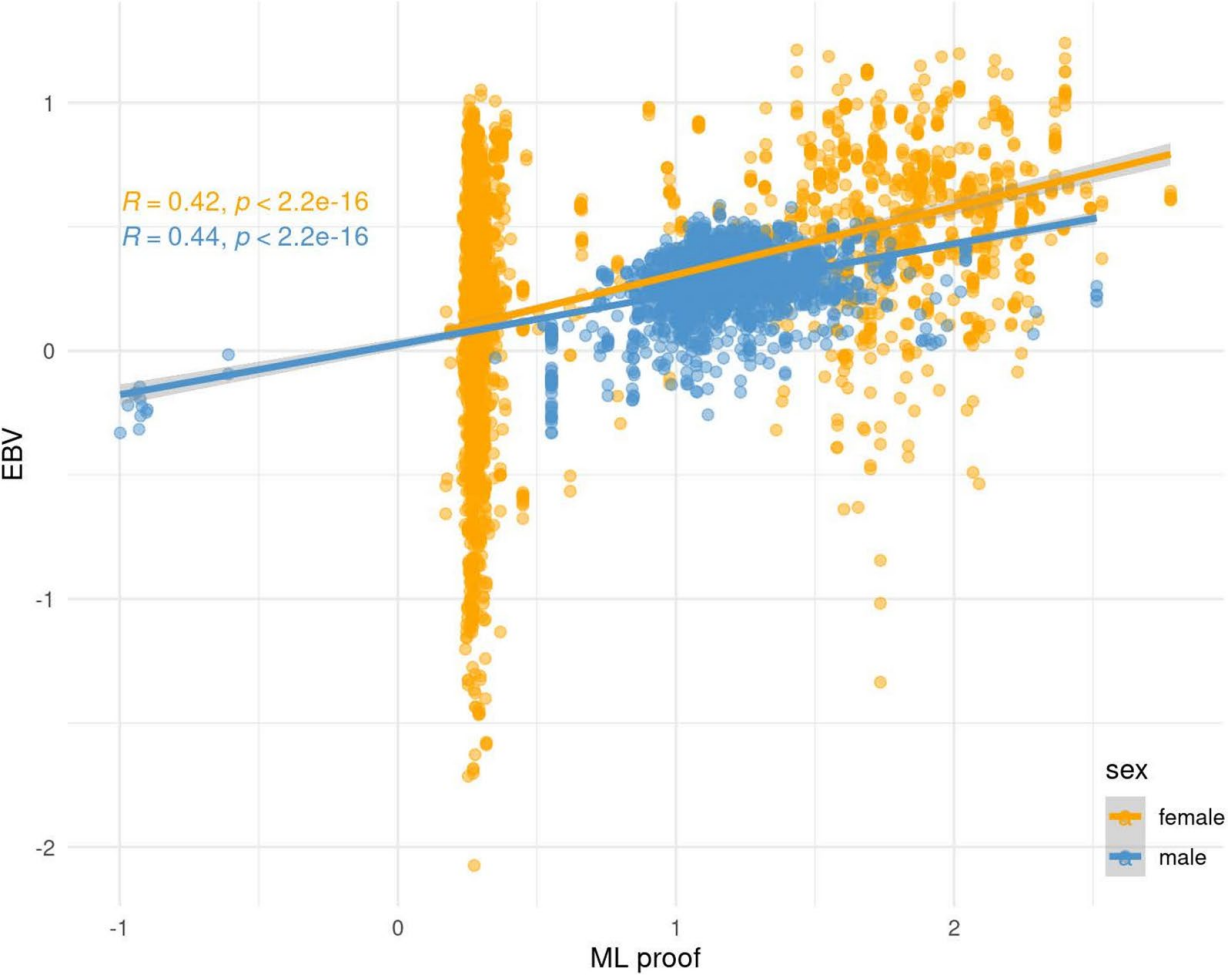
Estimated breeding values (EBVs) versus proofs produced from the two-tower MLP model using the final trained model and the full data as input. Color-coding is used for datapoints and regression lines corresponding to males and females. Pearson’s correlation coefficients and corresponding* p*-values are also printed with the same color-coding convention. Plot was created with ggplot2 v4.0.0 [27] and ggpubr v0.6.1 [30]

To assess ranking similarities between our two analytical approaches, we examined the relationship between EBVs and liabilities yielded from our ML model. Those seem to be significantly associated, (Fig. 3) with moderate correlation estimates of 0.42 and 0.44 for female and male respectively, although substantial dispersion remained. While both approaches use the same binary reproductive outcome and rely on pedigree-derived relationship information, they differ considerably in their modeling frameworks and assumptions. The Bayesian animal model estimates explicitly parameterized latent effects under pedigree-based covariance structures, priors and year effects, when the MLP learns nonlinear predictor combinations from founder-relationship features without explicit distributional assumptions or year adjustment. Because the true breeding values remain unknown, neither approach can be regarded as a reference standard. Consequently, the observed correlations indicate partial agreement in the ranking of individuals and suggest that both methods recover overlapping information about the same underlying latent genetic merit rather than producing directly interchangeable estimates.

## Conclusion

This study suggests that on-farm fertilization success can be modelled as a non-linear combination of latent fertility probabilities in a multiplicative fashion. Variance components were estimated under a Bayesian hierarchical framework suggesting moderate heritability for female fertility and a low-moderate estimate for male fertility in Icelandic Arctic charr. Additionally, we propose a flexible and easily implementable alternative based on a two-tower MLP architecture. Each tower models sex-specific liabilities, which are then integrated to predict cross outcomes (ROC AUC = 0.654). The framework is easily extendable to utilize multiple data modalities, including omics and diverse sources of metadata. From a practical standpoint, such models could support decision-making in animal production systems by assisting the identification of mating pairs with increased probability of reproductive success. However, in the current configuration, and given the moderate concordance between tower outputs and EBVs observed, tower outputs should be interpreted as predictive scores rather than direct substitutes for breeding values. In this proof-of-concept study, we evaluated the predictive ability of our ML system using only pedigree information as input. Future extensions could include genotypic information, effects from early embryo, compatibility effects between breeders and genotype-by-environment interactions (G × E) that are expected to be important for such traits.

## Supplementary Information

Supplementary Material 1.
Description : Diagnostic trace-plots for hierarchical Bayesian model.

Supplementary Material 2.
Description : Table with posterior summaries and diagnostic metrics for hierarchical Bayesian model.

Supplementary Material 3.
Description : Table with performance metrics from 4-fold cross-validation schemes to tune dropout rates.

## Data Availability

Data and code available at: https://github.com/pappasfotios/AC_Iceland_fertility.
