# Supplementary material for: A non-linear game for two: genetic parameters and prediction of fertilization success using Bayesian and machine learning frameworks
Source: Genet Sel Evol. 2026 Jul 16;58:34. doi: 10.1186/s12711-026-01070-9 (PMC13377850; doi:10.1186/s12711-026-01070-9)
Supplement: Supplementary file 1 — Supplementary Material 1. Description : Diagnostic trace-plots for hierarchical Bayesian model. [file 12711_2026_1070_MOESM1_ESM.pdf]

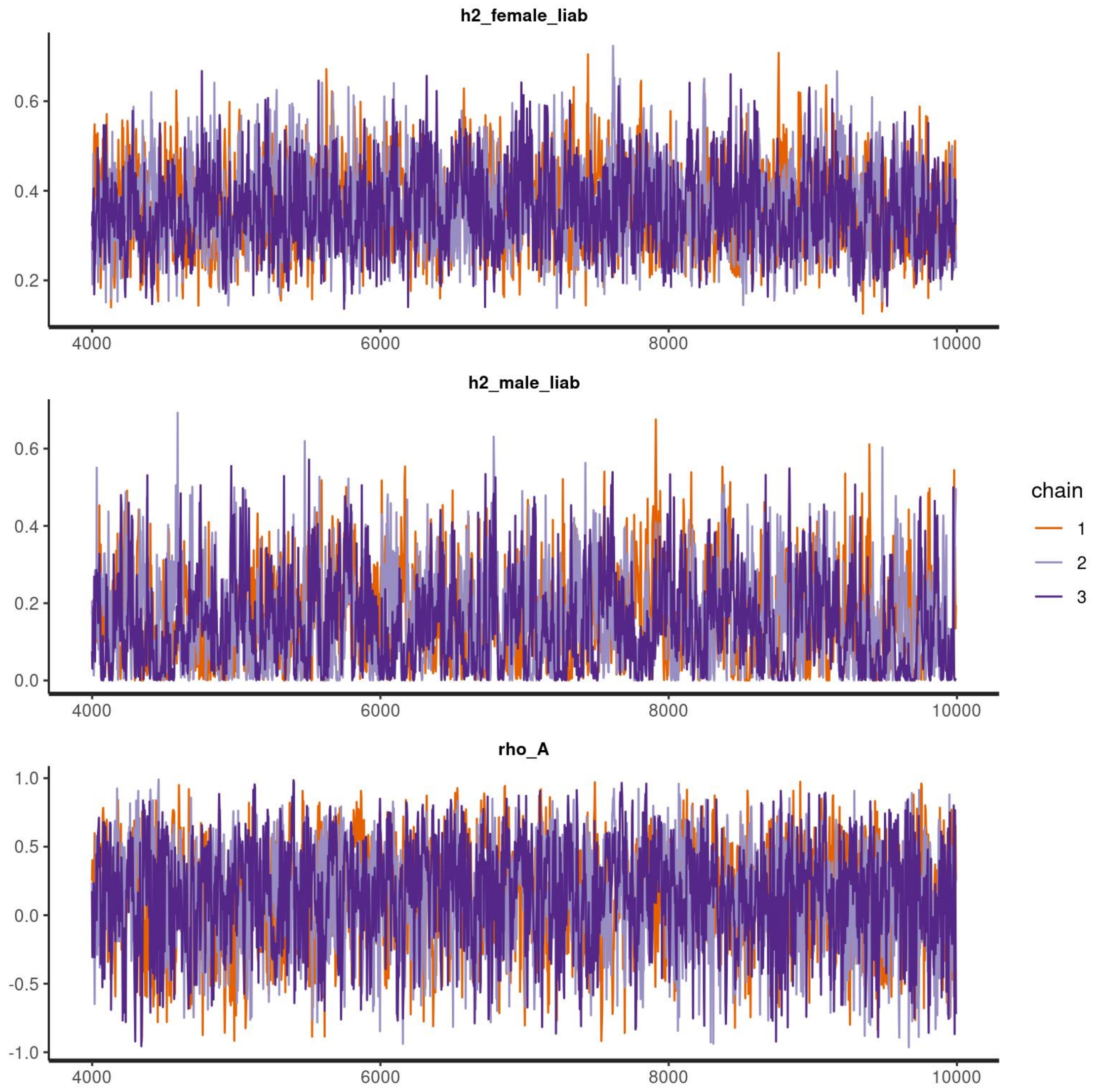

Figure S1: Trace-plots (only sampling iterations displayed) for male/female liability heritabilities and genetic correlation of the two.
