# Supplementary material for: A non-linear game for two: genetic parameters and prediction of fertilization success using Bayesian and machine learning frameworks
Source: Genet Sel Evol. 2026 Jul 16;58:34. doi: 10.1186/s12711-026-01070-9 (PMC13377850; doi:10.1186/s12711-026-01070-9)
Supplement: Supplementary file 2 — Supplementary Material 2. Description : Table with posterior summaries and diagnostic metrics for hierarchical Bayesian model. [file 12711_2026_1070_MOESM2_ESM.pdf]

Table S1: Posterior metrics and convergence diagnostics for intercepts, phenotypic variances, heritabilities and genetic correlation.

	**mean**	**sd**	**2.5%**	**97.5%**	$\hat{R}$	**N_eff**
**female intercept**	1.811	0.340	1.200	2.517	1.001	1833
**male intercept**	2.054	0.353	1.413	2.792	1.005	1627
σ_p^2 **female**	2.802	0.848	1.743	4.957	1.001	1566
σ_p^2 **male**	2.250	0.879	1.196	4.559	1.001	1294
h^2 **female**	0.363	0.093	0.199	0.556	1.001	1517
h^2 **male**	0.155	0.120	0.001	0.429	1.002	975
R_g	0.143	0.369	-0.639	0.784	1.000	2012

$\hat{R}$ is an indicator of chain-mixing upon approaching 1 while N_eff is the effective sample size of the Markov chain iterations.
