# Supplementary material for: A non-linear game for two: genetic parameters and prediction of fertilization success using Bayesian and machine learning frameworks
Source: Genet Sel Evol. 2026 Jul 16;58:34. doi: 10.1186/s12711-026-01070-9 (PMC13377850; doi:10.1186/s12711-026-01070-9)
Supplement: Supplementary file 3 — Supplementary Material 3. Description : Table with performance metrics from 4-fold cross-validation schemes to tune dropout rates. [file 12711_2026_1070_MOESM3_ESM.pdf]

**Table S2:** Sorted rid search results: ROC AUC and PR-AUC by dropout rate combination.

| Dropout 1 | Dropout 2 | ROC AUC | PR-AUC |
|---|---|---|---|
| 0.0 | 0.5 | 0.6454 | 0.9246 |
| 0.2 | 0.5 | 0.6437 | 0.9244 |
| 0.1 | 0.2 | 0.6435 | 0.9242 |
| 0.3 | 0.5 | 0.6423 | 0.9239 |
| 0.1 | 0.5 | 0.6414 | 0.9246 |
| 0.2 | 0.0 | 0.6411 | 0.9238 |
| 0.2 | 0.2 | 0.6410 | 0.9228 |
| 0.2 | 0.1 | 0.6405 | 0.9240 |
| 0.0 | 0.3 | 0.6403 | 0.9245 |
| 0.1 | 0.3 | 0.6400 | 0.9230 |
| 0.1 | 0.1 | 0.6397 | 0.9240 |
| 0.5 | 0.0 | 0.6388 | 0.9228 |
| 0.5 | 0.2 | 0.6386 | 0.9240 |
| 0.1 | 0.0 | 0.6383 | 0.9233 |
| 0.3 | 0.2 | 0.6381 | 0.9229 |
| 0.0 | 0.4 | 0.6381 | 0.9230 |
| 0.4 | 0.1 | 0.6375 | 0.9221 |
| 0.1 | 0.4 | 0.6375 | 0.9215 |

| Dropout 1 | Dropout 2 | ROC AUC | PR-AUC |
|---|---|---|---|
| 0.4 | 0.4 | 0.6369 | 0.9222 |
| 0.5 | 0.5 | 0.6369 | 0.9236 |
| 0.4 | 0.0 | 0.6367 | 0.9218 |
| 0.0 | 0.2 | 0.6367 | 0.9225 |
| 0.3 | 0.4 | 0.6366 | 0.9226 |
| 0.5 | 0.1 | 0.6365 | 0.9233 |
| 0.2 | 0.3 | 0.6357 | 0.9233 |
| 0.3 | 0.3 | 0.6355 | 0.9225 |
| 0.4 | 0.3 | 0.6352 | 0.9220 |
| 0.0 | 0.1 | 0.6352 | 0.9202 |
| 0.4 | 0.5 | 0.6352 | 0.9219 |
| 0.2 | 0.4 | 0.6344 | 0.9213 |
| 0.3 | 0.1 | 0.6338 | 0.9222 |
| 0.5 | 0.3 | 0.6337 | 0.9220 |
| 0.5 | 0.4 | 0.6307 | 0.9208 |
| 0.0 | 0.0 | 0.6303 | 0.9205 |
| 0.3 | 0.0 | 0.6293 | 0.9205 |
| 0.4 | 0.2 | 0.6262 | 0.9205 |
